# Multiple Bilateral Taurodontic Teeth in Primary Dentition: A Case Report

**DOI:** 10.5005/jp-journals-10005-1205

**Published:** 2013-08-26

**Authors:** Pallavi Vashisth, Swati Dwivedi, Satyaki Arora, Sandeep Mayall

**Affiliations:** Senior Lecturer, Department of Pedodontics, Institute of Dental Sciences Bareilly, Uttar Pradesh, India, e-mail: pallavivashisth@yahoo.in; Reader, Department of Pedodontics, Institute of Dental Sciences Bareilly, Uttar Pradesh, India; Senior Lecturer, Department of Pedodontics, Institute of Dental Sciences, Bareilly, Uttar Pradesh, India; Senior Lecturer, Department of Pedodontics, Teerthanker Dental College, Moradabad, Uttar Pradesh, India

**Keywords:** Bilateral, Multiple taurodontism, Deciduous teeth, Pulp chamber

## Abstract

Taurodontism describes the tendency for the body of the tooth to enlarge at the expense of the roots. An enlarged pulp chamber, apical displacement of the pulpal floor, and no constriction at the level of the cementoenamel junction are the characteristic features. These anatomic variations may hinder location of canal orifices, complete removal of pulp, proper instrumentation and obturation. Importance of radiographic interpretation for the diagnosis of this alteration cannot be overlooked. This article describes a case of multiple bilateral taurodontism involving all the deciduous molars in a 3½ year old female. The child reported with a chief complaint of multiple carious lesions.

**How to cite this article:** Vashisth P, Dwivedi S, Arora S, Mayall S. Multiple Bilateral Taurodontic Teeth in Primary Dentition: A Case Report. Int J Clin Pediatr Dent 2013;6(2):132-133.

## INTRODUCTION

The term ‘taurodontism’ was coined from the Latin term ‘tauros’, which means ‘bull’ and the Greek term ‘odus’, which means ‘tooth’.^1^ Although the condition was first described by Gorjanovic-Kramberger in 1908,^2^ the term was first proposed by Keith to describe molar trait seen in Neanderthal fossils. It describes the tendency for the body of the tooth to enlarge at the expense of the roots.^1^ Witkop defined taurodontism as ‘Teeth with large pulp chambers in which the bifurcations are displaced apically, so that the chamber has greater apico-occlusal height than in normal teeth and lacks the constriction at the level of the cementoenamel junction. The distance from the trifurcation and bifurcation of the roots to the CEJ is greater than the occlusocervical distance’.^3^

Taurodontism has been reported either as an isolated trait or as a feature of multiple system malformations syndromes, such as ectodermal dysplasia, Klinefelter syndrome, Down syndrome, tricho-dento-osseous syndrome and X-linked hypophosphatemic rickets.^4^ The trait can be seen in deciduous as well as permanent teeth or in both dentitions. Lysell reported a case in the deciduous second molars of a Swedish boy. The first molars did not show the trait. This is apparently the first reported case in deciduous teeth in modern man.^5^

This report describes a case of patient with multiple bilateral taurodontic teeth in deciduous dentition and the endodontic procedure undertaken.

## CASE REPORT

**Fig. 1:**
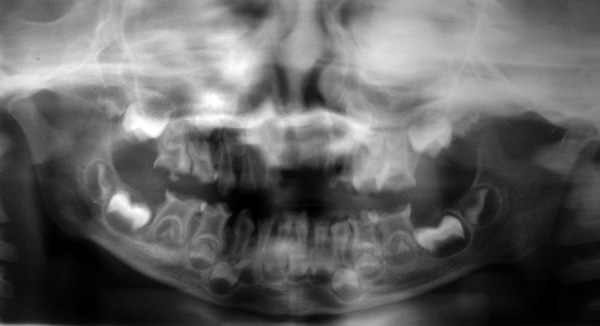
OPG of the patient showing multiple bilateral taurodontic teeth in maxillary and mandibular arch

A 3½ years old female patient reported to the Department of Pedodontics and Preventive Dentistry with multiple decayed teeth. Child's dental and medical history was not relevant. Soft tissue examination revealed no abnormality. Multiple carious lesions were seen with involvement of all the molar teeth. An orthopantomograph was advised, which revealed that all maxillary and mandibular molars were present with enlarged pulp chamber with short roots suggesting taurodontism (Fig. 1). No other obvious anomalies were noticed on the orthopantomograph. 74, 84 were classified as hypertaurodont and other molars were classified as hypotaurodont, based on the classification given by Shaw. Multivisit pulpectomy procedure was done in all the affected teeth. The presence of taurodontism was confirmed during the clinical procedure as the pulp chamber was large and filled entirely with pulp tissue. The pulp chamber and canals were debrided, shaped and obturated with Vitapex.

## DISCUSSION

Taurodontism is a dental anomaly characterized by large pulp chambers and short roots.^6^ Various etiological factors have been proposed for the development of taurodontism. Hammer proposed that developmental site of the taurodont is in Hertwig's epithelial root sheath and not in the odontoblast, since dentinogenesis of the root is not impaired. Taurodontism is caused by failure of Hertwig's epithelial sheath diaphragm to invaginate at the proper horizontal level.^7^

Mangion has proposed following as possible causes of taurodontism: A specialized or retrograde characteristic, a primitive pattern, Mendelian recessive trait. An atavistic feature, mutation resulting from odontoblastic deficiency during dentinogenesis of roots.^8^

First quantitative study on taurodontism was done by Shaw in 1928^9,10^ who classified taurodontism on the basis of relevant amount of apical displacement of the pulp chamber floor. The subtypes were hypomoderate enlargement of pulp chamber, meso pulp chamber enlarged and the roots short but still separate, hypertaurodont, where pulp chamber nearly reaches the apex and then breaks up into channels. Keene in 1966 has classified it on the basis of ‘Taurodont index’ (relative height of the pulp chamber to the length of the longest root).^10,11^

Taurodontism has been reported either as an isolated trait or as a feature of multiple system malformations syndromes.^4^ Sert and Bayrh reported a case of multiple taurodontic teeth without it being related to any syndrome.^12^The patient in our case had normal development of height– weight relationship with regard to the average percentile of children of her age. The patient's language and psychomotor skill development was consistent with her age. The case could not be associated to any syndrome.

Diagnosis is usually done by routine radiographs. From endodontist's view, taurodontism presents a challenge during negotiation, instrumentation and obturation in root canal therapy due to abnormal configurations of the root canal system, pulpal obliteration and increased tendency for additional root canals.^10^ Increase hemorrhage during access opening may be mistaken for perforation. Since, the roots are short and pulpal floor is placed apically, care should be taken to prevent perforation. In case of deciduous teeth conventional obturating materials like ZOE in such bulk may take longer time to resorb which may delay the natural exfoliation of the tooth.^13^ In the case of prosthetic treatment, it has been suggested that the placement of posts should be avoided in taurodontic teeth.

## CONCLUSION

As taurodonts show wide variations in size and shape of the pulp chamber with varying degrees of obliteration and canal configuration, root canal therapy becomes a challenge. The findings in this case strongly suggest the importance of radiographs in the diagnosis of alterations, such as taurodontism in which characteristics such, as multiple appearance and bilateral presence may appear.

## References

[1] Keith A (1913). Problems relating to the teeth of the earlier forms of prehistoric man. Proc R Soc Med.

[2] Gorjanovic-Kramberger K, Überprismatischemolar wurzeinre- zenter und diluvialer menschen (1908). Anatomischer Anzeiger.

[3] Witkop CJ Jr (1971). Manifestations of genetic diseases in the human pulp.. Oral Surg.

[4] Seow WK, Needleman HL, Holm IA (1995). Effect of familial hypophosphatemic on dental development: A controlled, longitudinal study.. Pediatr Dent.

[5] Lysell L (1965). Taurodontism in both dentitions. Report of a case.. Odontol Revy.

[6] Bharti R, Chandra A, Tikku AP, Wadhwani KK (2009). "Taurodontism" an endodontic challenge: A case report.. J Oral Sci.

[7] Hamner JE 3rd, Witkop CJ Jr, Metro PS (1964). Taurodontism: Report of a case.. Oral Surg Oral Med Oral Pathol.

[8] Mangion JJ (1962). Two cases of taurodontism in modern human jaws.. Br Dent J.

[9] Shaw JC (1928). Taurodont teeth in South African races.. J Anat.

[10] Jafarzadeh H, Azarpazhooh A, Mayhall JT (2008). Taurondism: A review of the condition and endodontic treatment challenges.. Int Endod J.

[11] Keene HJ (1966). A morphologic and biometric study of taurodontism in a contemporary population.. Am J Phys Anthropol.

[12] Sert S, Bayrl G (2004). Taurodontism in six molars: A case report.. J Endod.

[13] Bhat SS, Sargod S, Mohammed SV (2004). Taurodontism in deciduous molars: A case report.. J Indian Soc Pedod Prev Dent.

